# US state death rates: Structural equation modeling of Big Five personality, socioeconomic status, and health risk factors

**DOI:** 10.1177/13591053241306564

**Published:** 2025-01-02

**Authors:** Stewart JH McCann

**Affiliations:** Cape Breton University, Canada

**Keywords:** Big Five personality, biopsychosocial model, exercise, fruit and vegetable consumption, obesity, physical inactivity, sleep, smoking, socioeconomic status, US state death rates

## Abstract

Structural equation modeling (SEM) tested the *plausibility* of a *causal* model with neuroticism, openness to experience, socioeconomic status (SES), and race as predictors of a composite of six health risks and age-adjusted all-cause mortality in 2020 using the 48 contiguous American states as analytic units. In the final model, neuroticism, openness, and SES accounted for 80% of the health risk composite variance. These three variables and composite health risk accounted for 85% of the death rate variance. Neuroticism, openness, and SES had direct impacts on the health risk composite and indirect impacts on death rates through the health risk composite. SES and composite health risk also had direct impacts on death rates. Spatial autocorrelation and multicollinearity were not problematic. These SEM results underline the importance of state resident personality and SES in this context and support the *plausibility* of the *speculation* that the demonstrated relations may be *causal* in nature.

Does personality predict premature mortality? Empirical research with individuals as the analytic units strongly suggests that this is so. Big Five personality dimensions (e.g. John and Srivastava, 1999) have been related to all-cause mortality (e.g. Graham et al., 2017). As well, health risk factors such as lack of exercise, physical inactivity, lack of fruit and vegetable consumption, smoking, short sleep duration, and obesity predict death at an earlier age (e.g. Gale et al., 2017; Graham et al., 2017; Liu et al., 2021; Saint-Maurice et al., 2024; Silva et al., 2020; Visaria and Setoguchi, 2023). These risk factors also relate to the Big Five personality dimensions (e.g. Bogg and Roberts, 2004; Caille et al., 2024; Garrido et al., 2018; Zhang et al., 2024). Therefore, these findings also suggest the possibility of an indirect path from personality through health risk factors to age-adjusted all-cause mortality.

The “Big Five” is the most broadly accepted contemporary personality model. It includes five dispositional variables on which to differentiate persons within the nonpathological range of functioning—openness to experience, conscientiousness, extraversion, agreeableness, and neuroticism (e.g. John and Srivastava, 1999). Each dimension consists of six facets that relate to a number of trait descriptors. For example, the neuroticism facets are anxiety, angry hostility, depression, self-consciousness, impulsiveness, and vulnerability. They are associated with various trait adjectives such as being tense, irritable, discontented, shy, moody, and not self confident. In a similar manner, for example, ideas and actions facets of openness to experience are associated with being curious and having wide interests, competence and order facets of conscientiousness are associated with being efficient and organized, activity and warmth facets of extraversion are associated with being energetic and outgoing, and trust and compliance facets of agreeableness are associated with being forgiving and not stubborn (e.g. John and Srivastava, 1999). Each personality dimension also has a strong genetic component (e.g. Vukasović and Bratko, 2015).

Socioeconomic status (SES), largely based on income and education variables, generally is endorsed as a valid predictor of premature death. Individual-level research has firmly established that lower SES is associated with greater all-cause mortality (e.g. Glei et al., 2022). As well, lower SES has been associated with insufficient exercise, physical inactivity, lack of fruit and vegetable consumption, smoking, short sleep duration, and obesity (e.g. Ji et al., 2023; Murray et al., 2012; Stringhini et al., 2011). This also suggests an indirect path from SES through health risk factors to all-cause mortality.

Race also may predict mortality and health risk factors at the state level. For example, individual-level research has shown that being Black is associated with higher all-cause mortality (Gilmore et al., 2019) and with lack of exercise (e.g. Hersch, 1996), physical inactivity (e.g. Saffer et al., 2013), lack of fruit and vegetable consumption (e.g. Dubowitz et al., 2008), smoking (e.g. Persoskie and Leyva, 2015), short sleep duration (e.g. Nunes et al., 2008), and obesity (e.g. Lincoln et al., 2014). Perhaps there is a direct path to state death rates and an indirect path through health risk factors with Black population percent representing race at the state level.

Among the many risk factors for health and mortality, the six chosen for the present study were selected because they primarily reflect lifestyle. Other risk factors such as high blood pressure and high cholesterol were not included because they are not so directly driven by lifestyle choices. These six variables also have been used in many other studies and are appropriately documented for each state in annual reports (e.g. America’s Health Rankings, 2021).

The preceding research results linking all-cause mortality to Big Five personality, SES, and race are correlation-based, thus preventing causal inferences. However, there are reasons to believe the associations may be grounded in *causal* connections. For example, Kern and Friedman (2011) noted that personality can have an *impact* on health outcomes because it “influences the habits we form, the behaviors we engage in, the relationships we develop, our appraisals and experience of stressful challenges, the situations we commonly choose, the reactions we invoke in others, and the lifelong pathways that we follow” (p. 84), and of course, health outcomes clearly include health risks and ultimately death. Roberts et al. (2007) too suggested that personality may have causal *impacts* on mortality because personality differences may be related to pathogenetic mechanisms and because personality factors may foster differential reactions to illness. These somewhat interrelated paths of Kern and Friedman (2011) and Roberts et al. (2007) that eventually lead to death appear to stem *causally* from dispositional differences.

Relations of SES and race to mortality also seem rooted in *causal* relations. For example, causal mechanisms have been speculated to occur through SES-related variables such as diet quality, smoking, physical activity, and obesity (e.g. Laine et al., 2020). A review by Roberts et al. (2007) based on prospective longitudinal studies showed that the magnitude of SES effects on mortality essentially equaled those for personality. Regarding race, it is possible that states with higher Black population percents may have higher levels of risk factors and death rates caused by racial tendencies demonstrated at the individual level.

Several individual-level studies have found that relations of Big Five personality factors, SES, and race to all-cause mortality may be mediated through health risks (e.g. Grogan et al., 2024; Kim et al., 2018; Laine et al., 2020; Petrovic et al., 2018; Ploubidis and Grundy, 2009; Puka et al., 2023). For example, Grogan et al. (2024) conducted a literature review and reported that smoking consistently mediated the relation between neuroticism and mortality, and Laine et al. (2020) found that the relation of SES to all-cause mortality was mediated in part by smoking, physical activity, and obesity. Grogan et al. (2024) concluded that “the findings to date clearly show that mediation-based analysis has a key role to play in developing our understanding of the complex pathways that connect personality to mortality” (p. 222). Health risk factors also may mediate effects of SES (e.g. Kim et al., 2018) and race on mortality (e.g. Puka et al., 2023).

The present structural equation modeling (SEM) study uses American states as the cases. It is conducted from the geographical psychology perspective (e.g. Rentfrow et al., 2008; Rentfrow and Jokela, 2016). A major premise is that a geographic area’s aggregate position on an individual difference dimension relates to the level in that area of the behavioral and psychological tendencies associated with that individual difference dimension. Therefore, the approach provides a rationale for the *possibility* of prudently interpreting relations found at the aggregate level as stemming from processes occurring at the individual level. However, the approach recognizes that empirical support at each level eventually must buttress such speculations. Caution is necessary to avoid the pitfalls of the “compositional fallacy” (Pettigrew, 1997) and “ecological fallacy” (Robinson, 1950) which concern making mere *assumptions* regarding the validity of cross-level extrapolations.

State-level research from the geographical psychology perspective is beneficial. It can produce variables aggregated from individuals regarding psychological traits and processes that can be directly related to other state-level data produced by other government and private bodies. As well, geographical psychology with states as analytic units can present opportunities to evaluate the applicability of individual-level psychological findings at the state level, provide empirical findings with relevance for federal and state policy making, and furnish possibilities for evaluation of the effectiveness of public health policies and promotions (e.g. Chen et al., 2020).

The primary goal of the present research is to determine the extent to which state death rates can be predicted by selected state-level personality, sociodemographic, and health risk factors. The SEM analysis also provides a framework for judging the plausibility of expected direct and indirect *causal* paths from personality and demographic variables to health risk and to death rates. From the geographical psychology perspective, it is reasonable to *speculate* that such state-level relations may originate from parallel individual-level relations. Therefore, the present research also can be framed as a feasibility study to determine whether state-level relations within the current context resemble existing individual-level findings.

## The present study

The current state-level study uses SEM to test the *plausibility* of a *causal* model in which Big Five personality, SES, and race are expected to have direct effects on all-cause mortality as well as indirect effects on mortality through a composite of six major health risks. It was strongly expected that higher neuroticism, lower openness, and lower conscientiousness would be associated directly with higher composite health risk. It was less confidently expected that higher neuroticism, lower openness, and lower conscientiousness would be associated directly with higher death rates, but strongly expected that higher neuroticism, lower openness, and lower conscientiousness would be associated indirectly through health risks with higher death rates. Regarding SES, there were prime expectations that lower SES would be associated directly with higher composite health risk, that lower SES would be associated directly with death rates, and that lower SES would be associated indirectly through health risks with higher death rates. Regarding race, there were weak expectations that higher Black population percent would be associated directly with higher composite health risk, would be associated directly with higher death rates, and would be associated indirectly through health risks with higher death rates. Of course, it also was strongly expected that higher composite health risk would be associated directly with higher death rates.

## Method

### Measures

#### Death rate

Kochanek et al. (2023) provided the final age-adjusted death rate per 100,000 population in 2020 for each of the 50 American states. The state death rates were based on all races, both sexes, and all ages.

#### Big Five personality

Rentfrow et al. (2008) used the responses of 619,397 residents to the 44-item Big Five Inventory (John and Srivastava, 1999) administered online between December of 1999 and January of 2005 to create Big Five *z* scores for each of the 50 American states. They provided evidence that the sample generally was representative of the national population. They also indicated that they drew respondents from each state in direct proportion to the 2000 census figures regarding population and racial-ethnic composition, but the respondents were somewhat younger and more middle class than the state census estimates show. Rentfrow et al. (2008) also reported that each personality dimension had high temporal reliability based on two subsamples, a high mean inter-subsample correlation based on three random subsamples, a high congruence coefficient between state-level and individual-level factor structure, and high reliabilities with mean Cronbach alphas of 0.81 at the individual level and 0.89 at the state level.

#### SES

FRED (2024) provided the per capita personal income for each state in 2020. The US Census Bureau (2023) furnished the official state poverty rates for 2019 based on the American Community Survey, which it did not administer in 2020. The US Census Bureau (2024) supplied the percent in each state 25 years of age and over who had at least high school graduation and the percent who had at least a bachelor’s degree based on the American Community Survey for 2019. The sign was reversed for poverty rates (i.e. multiplied by −1). The standardized results of the sums of the four variables in *z* score form yielded an SES composite with a Cronbach alpha of 0.87.

#### Exercise

America’s Health Rankings (2021), based on 2019 data from the Behavioral Risk Factor Surveillance System (BRFSS), provided the percent of adults in each state who reported exercising enough to meet federal expectations. Those guidelines require “150 minutes of moderate or 75 minutes of vigorous aerobic activity and two days of muscle strengthening per week” (p. 99) within the previous 30 days. The state rates were reversed (i.e. multiplied by −1) for the present study.

#### Physical inactivity

America’s Health Rankings (2021) provided the percent in each state of “adults who reported doing no physical activity or exercise other than their regular job in the past 30 days” (p. 99) in 2020.

#### Fruit and vegetable consumption

America’s Health Rankings (2021) furnished the percent in each state of “adults who reported consuming two or more fruits and three or more vegetables daily” (p. 99) in 2019.

#### Smoking

America’s Health Rankings (2021) contributed the percent in 2020 in each state of “adults who reported smoking at least 100 cigarettes in their lifetime and currently smoke daily or some days” (p. 99).

#### Short sleep duration

America’s Health Rankings (2021) provided the age-adjusted percent of adults in each state who reported sleeping less than 7 hours per day in 2020. State residents responded to the following question asked through the BRFSS: “On average, how many hours of sleep do you get in a 24-hour period?” (p. 99).

#### Obesity

America’s Health Rankings (2021) also supplied the percent of adults 18 years and over in each state with a body mass index (BMI) of 30 or greater based on reported weight and height in 2020.

#### Race

KFF (2024) provided the Black population percent in each state in 2019. Figures were not available for 2020.

#### Health risk composite

A health risk composite was created based on the previously described exercise, physical inactivity, fruit and vegetable consumption, smoking, short sleep duration, and obesity variables using the data for the 50 states. Before computation, the exercise and fruit and vegetable consumption variables were reversed (i.e. multiplied by −1). Each of these six variables then was converted to z scores, the variables were summed, and the resulting score was standardized. This health risk composite variable has a Cronbach alpha of 0.91.

### Ethical considerations

All ethical standards were followed in all phases of this research. Formal ethics approval was not required because this article does not contain any studies with human participants performed by the author.

### Analytic strategy

Researchers employing SEM as a research tool do not discover or derive *causal* links *from* the SEM results. Instead, the SEM model tests whether the relations found in the data analyzed support the plausibility of *speculated* causal assumptions stemming “from the research design, prior studies, scientific knowledge, logical arguments, temporal priorities, and other evidence that the researcher can marshal in support of them” (Bollen and Pearl, 2013: 309). Fit statistics of SEM models serve as initial empirical tests of the plausibility of those assumptions based on the various elements of prior knowledge. Successfully “fitting the data does not ‘prove’ the causal assumptions, but it makes them tentatively more plausible” (Bollen and Pearl, 2013: 309).

The generated paths in the present SEM analysis begin with Big Five personality, SES, and race as primary exogenous variables with initially equal status in the potential to account for variance in health risks and mortality rates. It is important to know whether and to what extent common health risks may mediate the impacts of Big Five personality variables, SES, and race on all-cause mortality, and to do this without excessive statistical control of other variables that may be only marginally involved in the relations to which the present research has intentionally limited its analysis.

The present research focuses on state age-adjusted all-cause mortality rates in 2020. The AMOS SEM extension of SPSS 29 was used to determine the direct and indirect pathways to those mortality rates from SES, Big Five variables, and race using the health risk composite as the mediator. Only Big Five personality variables having a significant Pearson correlation with those mortality rates were included in the SEM analysis. Potential multicollinearity and spatial autocorrelation issues also were addressed.

## Results

Preliminary considerations led to the exclusion of Alaska and Hawaii, the two non-contiguous states of the USA. The main reason for excluding Alaska and Hawaii, which are quite distant from the other 48 states and from each other, was to facilitate the spatial autocorrelation analysis which requires states with borders touching other states. However, it also may be assumed that several apparent differences between these two states and the others might jointly contribute to distorted relations involving death rates and the potential predictors in the present study. Therefore, a screening for multivariate outliers was conducted using the SPSS Mahalanobis distance approach with the full 50 states. With death rate, each of the Big Five, SES, Black population percent, and the health risk composite in the variable pool, Alaska had the lowest Mahalanobis value of 27.35 (*p* = 0.001) and Hawaii had the second lowest value of 22.97 (*p* = 0.006). These results indicate that Alaska and Hawaii were multivariate outliers. This effectively constitutes another valid reason for basing the main analyses on only the 48 contiguous states.

Table 1 displays statistical means, standard deviations, and Pearson correlations based on the 48 adjoining states. Death rate significantly correlated −0.41 with openness, 0.43 with neuroticism, −0.81 with SES, between 0.58 and 0.83 with each of the six health risk variables, 0.50 with Black population percent, and 0.91 with the health risk composite.

**Table 1.** Means, standard deviations, and Pearson correlations for the 48 contiguous states.

Variable	*M*	SD	1.	2.	3.	4.	5.	6.	7.	8.	9.	10.	11.	12.	13.	14.
1. Death rate	861.51	104.93														
2. Openness to experience	−0.00	0.82	−0.41**													
3. Conscientiousness	0.11	0.89	0.22	−0.11												
4. Extraversion	0.01	0.97	0.16	−0.60***	0.36*											
5. Agreeableness	0.13	0.74	0.19	−0.34*	0.59***	0.54***										
6. Neuroticism	0.05	1.01	0.43***	0.07	−0.44**	−0.21	−0.27									
7. SES	−0.03	1.01	−0.81***	0.26	−0.40**	−0.12	−0.17	−0.25								
8. Lack of exercise	−22.48	3.27	0.75***	−0.43**	0.06	0.20	0.13	0.38**	−0.59***							
9. Physical inactivity	23.27	3.70	0.81***	−0.34*	0.12	0.17	0.01	0.54***	−0.80***	0.72***						
10. Lack of fruits/vegetables	−7.90	1.76	0.58***	−0.49***	0.32*	0.33*	0.20	−0.04	−0.51***	0.63***	0.51***					
11. Smoking	15.44	3.30	0.83***	−0.54***	0.11	0.17	0.16	0.36*	−0.67***	0.61***	0.70***	0.55***				
12. Short sleep duration	32.33	2.91	0.72***	−0.11	−0.05	−0.06	−0.14	0.58***	−0.64***	0.59***	0.78***	0.42**	0.53***			
13. Obesity	32.38	3.91	0.83***	−0.58***	0.22	0.27	0.32*	0.31*	−0.74***	0.70***	0.74***	0.53***	0.75***	0.62***		
14. Black population percent	10.60	9.49	0.50***	−0.03	0.11	−0.01	0.14	0.34**	−0.41**	0.28	0.51***	0.17	0.21	0.56***	0.47***	
15. Health risk composite	0.02	1.02	0.91***	−0.50***	0.16	0.22	0.14	0.42**	−0.79***	0.85***	0.89***	0.74***	0.84***	0.79***	0.87***	0.44***

* *p* < 0.05. **p < 0.01. ***p < 0.05.

### SEM models for the 48 contiguous states

#### Model 1

**Figure 1.**
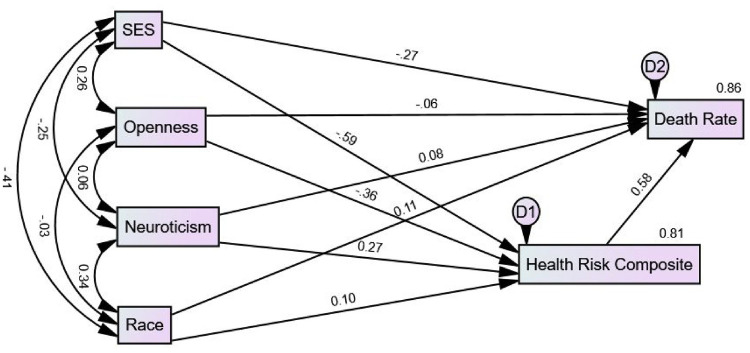
Saturated SEM Model 1 showing standardized path coefficients (βs) and *R*^2^ for SES, openness to experience, neuroticism, health risk composite, and death rate.

Figure 1 displays the standardized regression coefficients (βs) and the variance accounted for in the health risk composite and state death rates in the initial saturated model (Model 1). The β path coefficients were nonsignificant between death rate and openness (−0.06), neuroticism (0.08), and race (0.11). The path coefficients also were nonsignificant between the health risk composite and race (0.10). The *R*^2^ was 0.81 for the health risk composite and 0.86 for death rate.

#### Model 2

**Figure 2.**
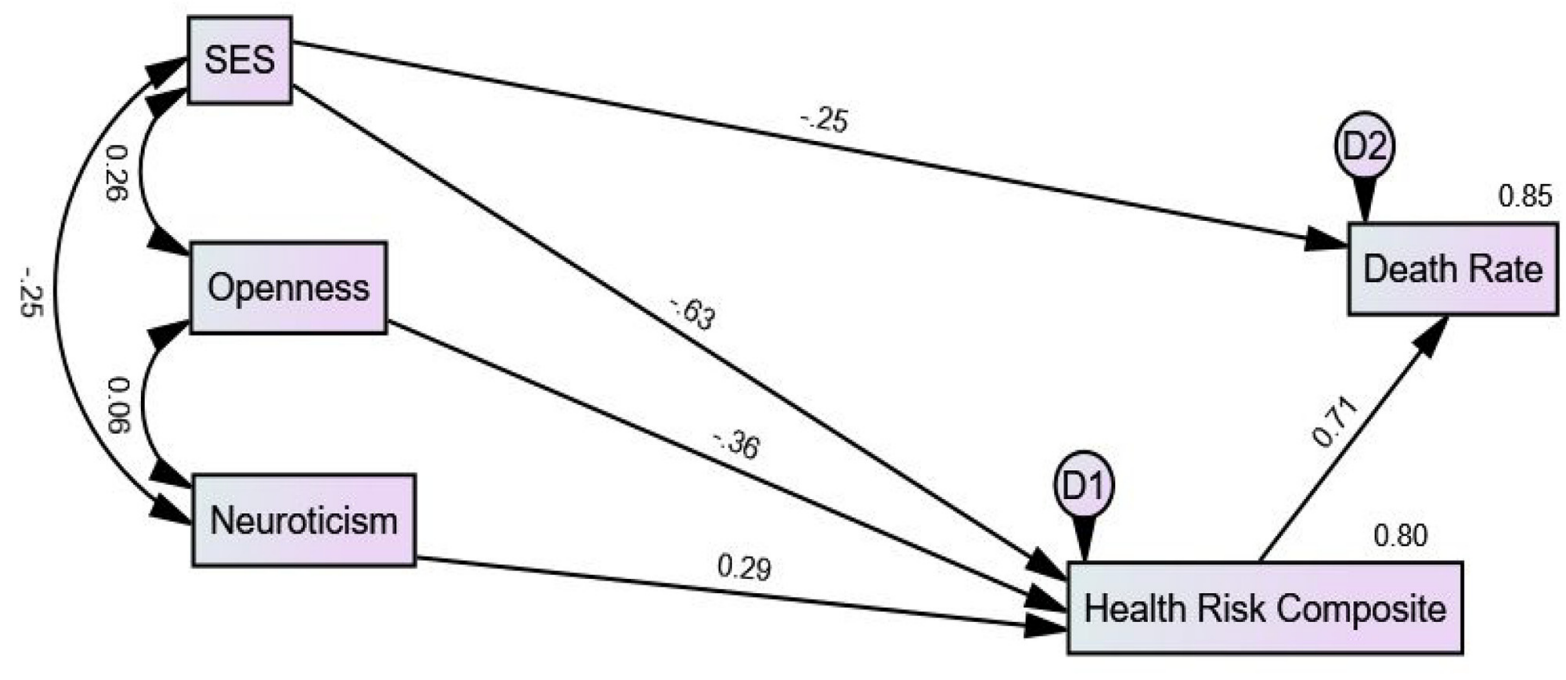
SEM Model 2 showing standardized path coefficients (βs) and *R*^2^ for SES, openness to experience, neuroticism, health risk composite, and death rate.

A reduced or overidentified Model 2 was developed with the four nonsignificant paths in Model 1 dropped (see Figure 2). Model 2 had an acceptable CMIN of 2.037 (df = 2; *p* = 0.361) indicating that the fit was not significantly poorer than that of Model 1. Other statistics showed a high goodness of fit of Model 2 to the data (e.g. RMSEA = 0.020; CFI = 1.000; Tucker-Lewis Index = 0.999; GFI = 0.983; SRMR = 0.0175). The β coefficient was significant and in the expected direction for the five direct paths: SES to the health risk composite (−0.63), openness to the health risk composite (−0.36), neuroticism to the health risk composite (0.29), SES to death rate (−0.29), and the health risk composite to death rate (0.71). The variance accounted for in Model 2 was 80% in the health risk composite and 85% in death rate, close to the values in Model 1.

Table 2 shows the standardized direct, indirect, and total effects in Model 2. Of course, the standardized direct effects in Table 2 are those previously reported for the direct paths in Model 2 (see Figure 2). The results for the standardized indirect effects showed that openness had an indirect effect through the health risk composite on death rate (−0.26), neuroticism had an indirect effect through the health risk composite on death rate (0.21), and SES had an indirect effect through the health risk composite on death rate (−0.45). The test of joint significance (Leth-Steensen and Gallitto, 2016) states that an indirect path is significant if the separate paths that constitute a compound path in an indirect effect are all significant. Therefore, these three indirect paths in Model 2 are significant. Standardized total effects simply are the sums of the standardized direct and indirect effects through the various paths in Model 2.

**Table 2.** SEM standardized direct, indirect, and total effects on the health risk composite and death rate in Model 2.

Standardized effects	Criterion	Openness to experience	Neuroticism	SES	Health risk composite
Direct	Health risk composite	−0.36	0.29	−0.63	0.00
	Death rate	0.00	0.00	−0.25	0.71
Indirect	Health risk composite	0.00	0.00	0.00	0.00
	Death rate	−0.26	0.21	−0.45	0.00
Total	Health risk composite	−0.36	0.29	−0.63	0.00
	Death rate	−0.26	0.21	−0.70	0.71

### Spatial autocorrelation and multicollinearity

SPSS 29 AMOS does not provide output regarding spatial autocorrelation. Therefore, the spatial autocorrelation analysis reported here is based on simultaneous ordinary least squares (OLS) multiple regression equations with death rate and the health risk composite as the criteria and their respective predictors taken from Model 1 and Model 2. For each of the four OLS equations, the global Moran’s I test identified spatial autocorrelation of residuals (Anselin, 2003) using the R *haven* and *spdep* statistical packages (R, 2022). To facilitate the analysis, the six variables were converted to *z* scores. A 48 × 48 Queen’s binary neighborhood matrix also was created that dummy coded each state touching another state at any point as a neighbor.

The four OLS equations first were tested for multicollinearity among predictors. All 16 VIFs were well below the common threshold of 10. Only one of 5.28 and one of 5.08 were 5.00 or above, and 13 were under 1.80. These results indicate that multicollinearity was not an issue with death rate or the health risk composite as the outcome variable.

With death age as the criterion, Moran’s I statistic standard deviate was 1.58 (*p* = .057) for Model 1 and 1.32 (*p* = .093) for Model 2. With the health risk composite as the criterion, Moran’s I statistic standard deviate was 1.63 (*p* = 0.052) for Model 1 and 1.30 (*p* = .096) for Model 2. These results indicate an absence of statistically significant spatial autocorrelation.

## Discussion

The final SEM model attests to the plausibility of a *causal* model in which higher neuroticism, lower openness to experience, and lower SES directly promote a composite of health risk factors that includes lack of exercise, physical inactivity, lack of fruit and vegetable consumption, smoking, obesity, and lack of sleep. A higher level of health risk factors then directly generates higher chances of premature age-adjusted all-cause mortality. Lower SES also has a direct impact on such mortality. This final model did not include either a direct positive impact of neuroticism on mortality or a direct negative impact of openness on mortality as was expected for the initial SEM model. However, neuroticism had an indirect positive impact while openness and SES had indirect negative impacts on mortality through the composite of health risk factors. Expectations regarding conscientiousness and race were not supported.

The present research employed American states rather than individuals as the analytic units. Therefore, it is important to compare these findings—at least for the direct effects—to previous individual-level findings to determine whether there is sufficient empirical evidence to justify *speculative* cross-level extrapolation. Generally, SES, openness to experience, and neuroticism have been related to health risk factors in the manner found in the present study (e.g. Caille et al., 2024; Garrido et al., 2018; Ji et al., 2023; Murray et al., 2012; Stringhini et al., 2011; Zhang et al., 2024). There also is individual-level evidence that each of the six risk factors and SES generally relate to all-cause mortality in the same way as in the present study (e.g. Glei et al., 2022; Liu et al., 2021; Saint-Maurice et al., 2024; Silva et al., 2020; Visaria and Setoguchi, 2023). Therefore, ample individual-level research demonstrates the relations of SES, openness, and neuroticism to health risk factors, and the relations of the six health risk factors and SES to all-cause mortality. These individual-level results provide corresponding corroborative evidence for the present state-level findings.

Neuroticism facets are associated with various trait adjectives such as being tense, irritable, discontented, shy, moody, and not self confident. In a similar manner, openness facets are associated with trait descriptors such as being curious, imaginative, artistic, excitable, and unconventional, and having wide interests. Accordingly, it would seem logical to assume that a neurotic person should be more likely to smoke, sleep poorly, make less than optimal food choices, exercise less, and engage in more sedentary behavior. It also would seem logical that an open person might be less likely to manifest such health risks. For example, being curious and having wide interests might make a person more open to engage in exercise, physical activity, diet modification, lifestyle modification to improve sleep, and efforts to refrain from smoking based on openness to pursuing and assimilating relevant new knowledge about these activities. Higher neuroticism or lower openness in a state also may contribute somewhat to relations between those two personality variables and health risk factors through social norms and expectations regarding health risks in part created and maintained through the presence of higher neuroticism and lower openness among state residents.

Although the current state-level Pearson correlations show that all-cause mortality related negatively to openness and positively to neuroticism in a statistically significant manner, neither Big Five variable retained a significant connection via a direct path to mortality in the SEM context. Many individual-level researchers have reported that higher neuroticism and lower openness are associated with a greater risk of all-cause mortality (e.g. Ferguson and Bibby, 2012; Grogan et al., 2024). The present significant Pearson correlations coupled with the nonsignificant SEM results suggests that the simultaneous control of other relations in the initial SEM model served to effectively reduce the direct relations of neuroticism and openness to all-cause mortality to nonsignificant levels. In fact, this was demonstrated in an unreported exploratory SEM model. Excluding the health risk composite yielded significant βs of −0.68 for SES, −0.26 for openness, and 0.27 for neuroticism.

The present state-level Pearson correlations of conscientiousness, extraversion, and agreeableness to all-cause mortality were not significant. Furthermore, none of these three significantly correlated with the health risk composite. In past individual-level research, these three personality variables have shown mixed relations to mortality (e.g. Bratt et al., 2016; Graham et al., 2017; Jokela et al., 2013). Given that the individual-level results are quite inconsistent, it may not be so unusual that statistically significant relations did not materialize here between these three personality variables and all-cause mortality.

However, of these three other Big Five variables, relations involving conscientiousness were most surprising in the present results. Conscientiousness correlated a nonsignificant 0.22 with both the health risk composite and death rate. These two *positive* correlations were unexpected and run counter to the *direction* of *most* research findings by others (e.g., Bogg and Roberts, 2004). Furthermore, with the 48 contiguous states as the analytic units, conscientiousness correlated a significant −0.40 with SES which also is in the unexpected direction (e.g. Conger et al., 2021). An exploratory partial correlation with SES controlled yielded nonsignificant *negative* correlations of −0.28 and −0.20 between conscientiousness and composite health risk and death rate, respectively. Why the existing state distribution patterns for conscientiousness and SES are in this way anomalous compared to most individual-level results reported by other researchers is unknown.

Although Black population percent correlated 0.44 with composite health risk and 0.50 with death rate, it was not significantly related to either criterion in the SEM analysis. This lack of relations may have occurred in part because of the correlation of −0.41 between race and SES. An unreported exploratory partial correlation with SES as the control yielded a nonsignificant correlation of 0.21 between race and composite risk, while another exploratory partial correlation with composite risk controlled produced a nonsignificant correlation of 0.28 between race and death age. These relational patterns evidently contributed to the elimination of Black population percent in the SEM analysis.

Each of the six health risks used in this study to create the composite seem to represent the opposite of what throughout most of human history would have been considered assets for coping with the demands of a successful existence—that is, being relatively physically fit, having an adequate diet, getting enough sleep, and avoiding the intake of foreign substances that are neither edible nor potable and may be detrimental to health. Therefore, it does not seem so surprising that geographical environments such as American states in which these health maintaining behaviors are practiced to a lesser degree also tend to have higher age-adjusted all-cause death rates. It also does not seem so surprising that American states with lower SES, higher neuroticism, and lower openness tend to participate less in such health maintaining behaviors and that ultimately leads to a greater chance of dying from a variety of causes. In addition to these paths of influence on state death rates, lower SES also signifies other behaviors, beliefs, attitudes, and values that may lead to premature mortality. The present SEM results support these links and bolster the plausibility that they are causal in nature.

### Limitations and issues

Some readers might assume that the present research seriously suffers from the detrimental effects of the small *sample* size of 48 states. However, the *sample* here also is the defined *population*. Descriptive statistics apply equally to the population and the sample. Logically, this makes inferential statistics unnecessary. Nevertheless, they have been included here to satisfy conventional reporting practice, facilitate decisions in the analytical process, and provide standard benchmarks for comparison purposes. Analytic strategies based on correlation, multiple regression, and SEM often have been used successfully with similarly sized samples (e.g. McCann, 2008; Sideridis et al., 2014).

Another issue that might arise has to do with the fact that the state Big Five personality scores developed by Rentfrow et al. (2008) were based on data collected from 1999 to 2005, well before the current focus year of 2020. However, there are several reasons to allay such a concern. For example, Elleman et al. (2018) demonstrated that such state scores were temporally stable and maintained consistent relations with sociodemographic variables from 1999 to 2015—the range of years they analyzed. Studies using other sociodemographic variables measured more recently also have employed these state scores successfully (e.g. McCann, 2023; Webster et al., 2023).

This is a cross-sectional study with analytical techniques grounded in correlations. Strictly speaking, the usual caution that causality cannot be inferred from correlational evidence must be applied. However, a *speculated* causal model for the associations between variables was tested for *plausibility.* That analysis produced evidence supporting the view that the paths between variables in the model—both direct and indirect—indeed may be *causal* links. Nevertheless, the model coefficients cannot be interpreted as sufficient evidence to unequivocally assert that such causal connections actually exist in the state-level data.

Other factors also may play roles in relation to health risks and all-cause mortality. For example, personality dimensions outside of the current framework such as impulsivity (Kale et al., 2018), sensation seeking (De Moor et al., 2006), and honesty-humility (MacCann et al., 2015) also have been implicated regarding health risks. As well, it is important to acknowledge that structural and systemic factors related to racism (e.g. Murry et al., 2024) and sexism (e.g. Homan, 2024) also may have an impact on SES, health risk factors, premature mortality, and perhaps even personality differences to some degree. The extent to which such excluded factors may have an impact on the present SEM analysis is, of course, unknown.

### Potential implications for application

The present study was primarily basic research to enhance our understanding of the relations between personality, health risks, and mortality with states as the analytic units. However, several aspects of the results also hold *potential* implications for application. The SEM analysis suggests that states with higher age-adjusted all-cause death rates should focus efforts to reduce these mortality rates on the reduction of the six health risk variables in the health risk composite and on the raising of SES. Each of the health risk variables and the education and income components of SES are potentially modifiable. Information and incentive campaigns directed at state residents to increase the percentage engaged in the six health risk variables and the pursuit of more education and higher incomes may eventually lead to better population health and lower rates of premature mortality.

The final SEM model also suggests that openness and neuroticism have direct impacts on health risks as well as indirect impacts through those health risks on death rates. However, state differences on these two dispositional variables are largely rooted in state gene pools, firmly integral to the person, and quite stable throughout life. They are not readily, if at all, responsive to intentional modification efforts. Nevertheless, information and incentive campaigns to encourage a healthier lifestyle might have greater success if they were tailored to appeal to those who are low on openness and to those high on neuroticism depending upon state levels of those two personality variables. For example, such tailoring approaches based on Big Five differences have been put forward by Olsacher et al. (2023) to encourage organ donation in Germany and by Kim et al. (2023) for the provision of optimal health care.

### Future research

The present SEM results were obtained with states as the analytic units. Ample individual-level research has been cited to support the *speculation* that direct and indirect paths involving SES, openness, neuroticism, health risks, and all-cause mortality may stem from parallel paths with individuals as the analytic units. Therefore, justification of such cross-level extrapolation would be on even firmer ground if the same SEM results pattern could be demonstrated in a future *individual-level* replication using the same variables.

Whether the present aggregate-level SEM results based on American states generalize to other nations and cultures also deserves research attention. For example, the UK has readily available Big Five personality scores for 380 local authority districts (LADs; Rentfrow et al., 2015). As well, socioeconomic grade data also are available for each LAD (UK Geographics, 2014). Data for all-cause mortality and each of the six health risk variables also are likely to be obtainable from various UK sources. Therefore, an initial cross-cultural UK SEM analysis seems feasible using LADS as the analytic units. Other countries also may afford such replication opportunities.

## Conclusion

The present SEM results based on the 48 contiguous American states as analytic units shows that 85% of the variance in age-adjusted death rates can be accounted for by four variables—SES, openness to experience, neuroticism, and a health risk composite derived from lack of exercise, physical inactivity, lack of fruit and vegetable consumption, smoking, obesity, and lack of sleep. As well, 80% of the variance in the health risk composite scores can be accounted for by three variables—SES, openness to experience, and neuroticism. States with lower SES, lower openness, higher neuroticism, and higher health risk composite scores have higher death rates. States with lower SES, lower openness, and higher neuroticism have higher health risk composite scores. SES has a direct impact on death rates and an indirect impact through health risks on death rates. Openness to experience and neuroticism have indirect impacts through health risks on death rates.

The results underline the importance of state socioeconomic variables *and* resident personality to explaining state differences in health risks and age-adjusted death rates. The SEM results also support the plausibility of the *speculation* that the demonstrated relations are *causal* in nature. As well, with due caution, it is *speculated* that the present state-level results stem from parallel relations at the individual level. Generally, the present research also demonstrates the usefulness of the geographical psychology approach for further understanding of population health.
